# Experimental Study on the Preparation of Paste Filling Materials from Coal-Based Solid Wastes

**DOI:** 10.3390/ma18143244

**Published:** 2025-07-09

**Authors:** Chaowen Hu, Xiaojie Yang, Feng Zhang, Bo Pan, Ruifeng Huang, Bing Hu, Yongyuan Li, Lei Zhang, Bingshan Wang, Jianxun Gao, Huifeng Wang, Yun Yu

**Affiliations:** 1 Huaneng Coal Technology Research Co., Ltd., Beijing 100070, China; 18518556655@163.com (B.P.); 13810603612@163.com (B.H.); 15203308090@163.com (Y.L.); 13165479123@163.com (L.Z.); 18612568551@163.com (B.W.); 13001100105@163.com (J.G.); 18681930371@163.com (H.W.); 2 China Coal Technology and Engineering Group, Chongqing Research Institute, Chongqing 400037, China; zfhxq1030@163.com; 3 State Key Laboratory for Tunnel Engineering, China University of Mining and Technology, Beijing 100083, China; 4 School of Emergency Technology and Management, North China Institute of Science and Technology, Beijing 101601, China; hrfcumt@163.com; 5 Huaneng Coal Co., Ltd., Beijing 100070, China; 13001152093@163.com

**Keywords:** mining under structures, coal-based solid waste, composite cementitious backfill materials, land subsidence

## Abstract

To reduce the cost of coal mine filling materials, a novel composite cementitious material was developed by utilizing coal-based solid waste materials, including fly ash, desulfurized gypsum, and carbide slag, along with cement and water as raw materials. Initially, a comprehensive analysis of the physical and chemical properties of each raw material was conducted. Subsequently, proportioning tests were systematically carried out using the single-variable method. During these tests, multiple crucial performance indicators were measured. Specifically, the fluidity and bleeding rate of the slurry were evaluated to assess its workability, while the compressive strength and chemically bound water content of the hardened sample were tested to determine its mechanical properties and hydration degree. Through in-depth analysis of the test results, the optimal formulation of the composite cementitious material was determined. In the basic group, the mass ratio of fly ash to desulfurized gypsum was set at 70:30. In the additional group, the carbide slag addition amount accounted for 20% of the total mass, the cement addition amount was 15%, and the water–cement ratio was fixed at 0.65. Under these optimal proportioning conditions, the composite cementitious material exhibited excellent performance: its fluidity ranged from 180 to 220 mm, the bleeding rate within 6 h was less than 5%, and the 28-day compressive strength reached 17.69 MPa. The newly developed composite cementitious material features good fluidity and high strength of the hardened sample, fully meeting the requirements for mine filling materials.

## 1. Introduction

China is abundant in coal resources. However, continuous mining has led to the increasing prominence of the issue of coal mining under buildings, railways, and water bodies. Statistics indicate that the reserves of coal affected by these pressures in China exceed 14 billion tons, accounting for 63.5% of the country’s recoverable coal reserves [1,2,3]. Consequently, the scientific and safe extraction of these overburdened coal resources has become a focal point in the coal mining industry. Currently, backfill mining, a method that controls strata movement by filling goafs with backfill materials, has proven to be an effective solution for the issue of coal mining under buildings, railways, and water bodies [4,5,6]. After years of development, backfill mining technology has matured, and backfill materials have evolved into three major categories: inert backfill materials, cemented backfill materials, and new functional backfill materials. Inert backfill materials encompass gangue, river sand, loess, etc. Cemented backfill materials include paste backfill materials, industrial waste residue cemented materials, and others. New functional backfill materials consist of high-water quick-setting backfill materials, fire-prevention and fire-fighting backfill materials, polymer materials, and subsidence-reducing backfill materials [7,8,9,10]. In recent years, extensive research on the characteristics of backfill materials has been conducted by scholars both domestically and internationally. Academician Xie Heping’s team has introduced a novel concept and technical framework for carbon-negative backfill mining. They have established a structural design for carbon-negative, highly porous backfill materials composed of a mixture of CO_2_, gangue, and rapid-setting cementing agents. By improving and designing a closed carbon-sequestering stirring reactor, wet-stirring carbon-sequestration tests were conducted to investigate the carbon-sequestration performance of coal gangue solid waste. The correlation between carbon-sequestration performance and factors such as stirring speed and reaction temperature was analyzed, providing theoretical support for the design and application of carbon-negative backfill materials and promoting the implementation of the “double carbon” goal in the coal industry [11]. To address the air leakage problem around gas drainage boreholes, a relevant research team has developed a novel sealing material for dynamic filling and plugging of fractures. This study tested and compared the material’s rheological properties, permeability, and stability. The experimental results demonstrated that the new material exhibits superior performance [12]. Based on the characteristics of hydration heat and volume resistivity, this study thoroughly investigates the hydration kinetics process and microstructural formation mechanism of fresh fly-ash cemented backfill materials. Using multiple testing methods, key nodes in the material hydration process are identified, and the evolution law of the microstructure is analyzed. This research is of great significance for reasonably regulating the performance of backfill materials [13]. Yin Bo et al. [14] focused on fly-ash cemented backfill materials and studied the enhancement of their strength and deformation properties through electrochemical treatment. The paper elaborated on the experimental procedures, analyzed the microstructural changes in the treated materials, and revealed the internal mechanisms underlying the performance enhancement. This work provides a new approach for optimizing the properties of backfill materials.

Although many scholars have developed various backfill materials with excellent mechanical properties, reducing the cost of backfill materials remains a key concern in the coal mining industry. This research aimed to reduce the cement consumption in traditional cemented backfill materials and break through the technical bottleneck of high backfilling costs. Meanwhile, by following the path of solid waste resource utilization, this study achieved the collaborative underground disposal of industrial wastes such as fly ash, desulfurized gypsum, and calcium carbide sludge, forming a green backfill technology system of “treating waste with waste and turning waste into treasure.” This system provides theoretical foundations and technical support for the safe and efficient mining of coal resources and ecological environmental protection in coal mines. A novel composite cementitious backfill material was developed, primarily using industrial solid wastes such as fly ash, desulfurized gypsum, and carbide sludge. Single-variable method was employed to complete the proportioning tests of the composite cementitious material, and the fluidity and water bleeding rate of the slurry, as well as the compressive strength and chemically bound water content of the solidified body, were tested. XRD and SEM techniques were utilized to analyze the crystal phases of hydration products and the micro-morphology of the composite cementitious material, enabling an in-depth investigation of its hydration process and mechanism.

## 2. Materials and Methods

The raw materials used in this study include fly ash, desulfurized gypsum, calcium carbide slag, and cement. Fly ash is a fine powder collected from the flue gases of coal-fired power plants and is the main solid waste discharged from coal-fired power plants, with major components including SiO_2_, Al_2_O_3_, and Fe_2_O_3_. Desulfurized gypsum is an industrial by-product generated during the flue gas desulfurization process in coal-fired power plants or steel mills, with the main component being CaSO_4_ 2H_2_O. The fly ash and desulfurized gypsum used in the experiments were sourced from a local coal-fired power plant. Calcium carbide slag is an industrial waste residue from the production of acetylene by the hydrolysis of calcium carbide (CaC_2_), with the main component being calcium hydroxide (Ca(OH)_2_). The calcium carbide slag used in the experiments was sourced from a local PVC plant. The cement used in the experiments was ordinary Portland cement produced by a local cement factory, with major chemical components including Al_2_O_3_, SiO_2_, and CaO.

To ensure that the mechanical properties of the composite cemented backfill material meet the on-site engineering requirements, it is necessary to determine the optimal mix proportion of the composite cemented backfill material through tests such as slurry fluidity, water separation rate, compressive strength of the hardened sample, and chemically bound water content. The performance of the composite cemented backfill material is analyzed via crystalline phase and micro-morphology tests of the hydration products.

Due to the similar state between the slurry of fly ash composite cementitious material and cement mortar, the fluidity of the slurry can be measured by referring to the *Test Method for Fluidity of Cement Mortar* (GB/T 2419-2005) [15]. The bleeding rate of the slurry should be determined according to the *Technical Specification for Application of Cementitious Grouting Materials* (GB/T 50448-2015) [16]. The compressive strength of the solidified body shall be tested in accordance with the *Test Method for Strength of Cement Mortar (ISO Method)* (GB/T 17671-2021) [17]. The chemically bound water content of the solidified body shall be measured as specified in the *Methods of Chemical Analysis of Cement* (GB/T 176-2017) [18].

### 2.1. Experimental Investigation into the Mix Proportion Optimization of Composite Cemented Backfill Materials

#### 2.1.1. Slurry Fluidity

To facilitate pipeline transportation and ensure efficient backfilling, the composite cementitious material must exhibit optimal fluidity, with its flowability maintained within the range of 180–240 mm. Given that the slurry of fly-ash composite cementitious material closely resembles cement mortar in consistency, a cement mortar flowability tester is suitable for measuring its flowability [19,20]. Flowability is quantified as the average diameter of the cement mortar spread on a flow table. A larger average diameter indicates higher flowability and, consequently, better fluidity [21,22].

#### 2.1.2. Slurry Water Separation Rate

The water separation rate of the slurry is a critical performance index that reflects its stability. A lower water separation rate indicates better stability. Generally, a slurry is defined as stable if its 6-h water separation rate is less than 5% [23,24]. The 6 h water separation rate is commonly used as an indicator to evaluate the stability of slurries. The test method for determining the water separation rate is as follows [25,26]: A 500 mL graduated cylinder is filled with approximately 400 mL of the slurry, and the initial slurry volume is recorded as V_1_. After standing undisturbed for 6 h, the volume of separated water above the slurry is measured and recorded as V_2_. The water separation rate α is calculated as the ratio of V_2_ to V_1_.

#### 2.1.3. Compressive Strength of the Hardened Sample

Uniaxial compressive strength tests were performed on the hardened sample [27,28]. Specimens were cast using three-joint mortar molds with dimensions of 70.7 × 70.7 × 70.7 mm. After demolding, the specimens were cured in a standard cement constant temperature and humidity chamber. Compressive strengths were measured at ages of 3, 7, and 28 days. For each test group, three specimens were tested, and the average value of their compressive strengths was regarded as the test result of the group, accurate to 0.01 MPa. In case the compressive strength reading of an individual specimen deviated significantly (±20%) from the others, it was excluded, and the average value was recalculated based on the remaining specimens. The loading rate applied during the compression tests was set at 2.4 kN/s, in accordance with the relevant testing standards, to ensure consistent and comparable results.

#### 2.1.4. Chemically Bound Water Content in the Hardened Sample

In this study, the hydration degree of the composite cementitious material is characterized by its chemically bound water content, which is determined using the ignition loss method [29,30]. The specific procedures are as follows: Initially, the sample is ground into a fine powder with an average particle size of 80 μm to terminate the hydration process, typically by soaking in absolute ethanol. This particle size control ensures consistent reaction kinetics and uniform termination of the hydration process. Next, 3 g of the ethanol-treated powder is placed in a pre-weighed crucible. The crucible is then dried at 105 °C until a constant weight is achieved. The sample is removed, cooled to room temperature, and weighed as m_105_. Subsequently, the sample is transferred to a muffle furnace, heated from room temperature to 950 °C, and held at this temperature for 20 min. After cooling in a desiccator to room temperature, the sample is weighed again as m_950_. The chemically bound water content is calculated using the following formula [31,32]:

(1) $$w=\frac{m_{105}-m_{950}}{m_{105}}\times 100\%-\left(1-\alpha -\beta \right)L_{1}-\left(1-\alpha \right)L_{2}-\beta L_{3}$$

where: W—Chemically bound water content, (%); m_105_—Mass of the sample after drying at 105 °C and cooling to room temperature, (g); m_950_—Mass of the sample after drying at 950 °C and cooling to room temperature, (g); L_1_, L_2_, L_3_—Ignition loss of cementitious materials, (%); α,β—Proportions of cementitious materials α and β in the total amount of cementitious materials, (%).

### 2.2. Performance Evaluation of Composite Cemented Backfill Materials

The crystalline phase composition and structure of hydration products are key factors that determine the performance of mine backfill materials. The micro-morphology of these hydration products directly influences the engineering properties of composite cemented backfill materials, including mechanical strength, durability, permeability, and stability. Therefore, following the determination of the mix proportion of the composite cemented backfill material, its performance is evaluated by analyzing the crystalline phase and micro-morphology of its hydration products [33].

#### 2.2.1. Crystalline Phases of Hydration Products

In this study, X-ray diffraction (XRD) tests are employed to analyze critical parameters such as the crystalline state and changes in the crystal structure of hydration products in cementitious materials [34,35]. The specific procedures are as follows: Prior to testing, the sample is vacuum-dried to eliminate moisture interference. The sample is then ground into a fine powder with a particle size distribution of D90 < 20 μm (verified by laser diffraction analysis), and 3 g of the powder is weighed to prepare a 10 mm × 10 mm thin slice. The powder is compacted under 10 MPa to minimize preferred orientation effects, following the protocol of ASTM C1365-18. The X-ray source is a rotating anode with a vibration amplitude controlled below 0.125 mm. The light spot focuses from 0.04°, with a focusing range of 0.25 mm × 15 mm. The copper tube provides a maximum voltage of 45 kV and a maximum rated current of 40 mA. The scanning equipment consists of a 2θ rotating goniometer driven by a motor, positioned vertically with a maximum speed of 1600°/min. The slit system includes a Soller slit (15 mm), a divergence slit (0.12 mm), a receiving slit (7.5 mm), and an anti-scatter slit (0.2 mm). The sample measurement angle ranges from 4° to 70°. The test results are analyzed and processed using JADE 6.5 software.

#### 2.2.2. Micro-Morphology of Hydration Products

The micro-morphology of hydration products directly influences the engineering properties of composite cementitious backfill materials, including mechanical strength, durability, permeability, and stability [36,37]. In this study, a GAIA3-type dual-beam field emission scanning electron microscope (FESEM, TESCAN Orsay Holding, a.s., Brno, Czech Republic) equipped with an X-ray diffractometer (D8 ADVANCE, Bruker Corporation, Billerica, MA, USA) was used to examine the micro-morphology of hydration products at curing ages of 3, 7, and 28 days. The EDS analysis was performed under the following conditions: an accelerating voltage of 15 kV, a working distance of 10 mm, an acquisition time of 100 s, and a beam current of 1.2 nA. Prior to testing, samples were pretreated as follows: 1 g of each sample was crushed (rather than ground, to preserve the crystal structure) and immersed in absolute ethanol to terminate the hydration reaction and to disperse the sample. A droplet of the dispersed sample was then placed onto the conductive adhesive using a dropper. After the ethanol evaporated, the conductive adhesive strips with attached samples were subjected to gold sputtering using an ion sputtering instrument. Prepared samples were stored in a sealed container to prevent contamination.

#### 2.2.3. Field Test

The overburden surface of the #3 coal seam in Xinyuan Coal Mine is occupied by Chenjiagou Village. The requirement for the holistic relocation of Chenjiagou Village to facilitate the mining of the #3 coal seam has significantly increased the operational costs associated with coal resource extraction. To address this challenge, this study explores the development of composite cementitious backfill materials derived from coal-based solid waste. The research aims to ensure that surface subsidence remains within the permissible limits set by industry standards, while simultaneously reducing backfilling costs to enable safe and economically viable exploitation of coal resources in the #3 coal seam.

**Figure 1:**
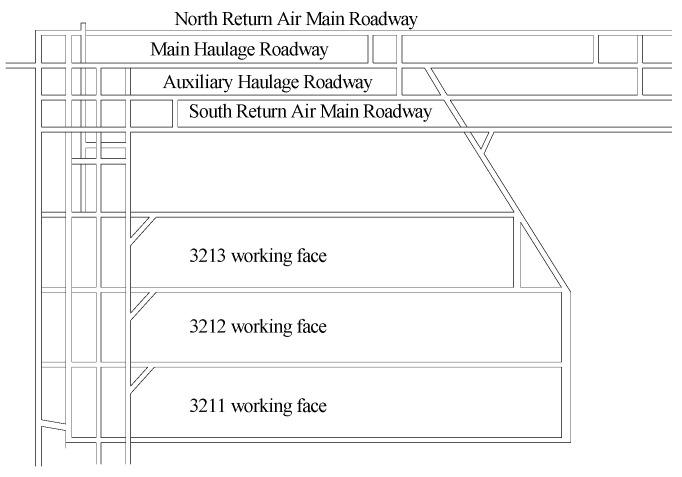
The layout of these test working faces.

Three short-wall working faces (3211, 3212, and 3213) in the #3 coal seam were selected as test sites. Each working face has a dip length of 65 m and a strike length ranging from 340 to 390 m. The layout of these test working faces is depicted in Figure 1.

## 3. Results and Discussion

### 3.1. Mix Proportion Optimization of Composite Cementitious Backfill Materials

#### 3.1.1. The Ratio of Fly Ash to Desulfurized Gypsum

The dosage of carbide slag was set at 15%, cement at 10%, and the water-to-cement ratio at 0.65. Five experimental groups were designed with varying ratios of fly ash to desulfurized gypsum: 50:50, 60:40, 70:30, 80:20, and 90:10. The test proportions for the influence of fly ash to desulfurized gypsum ratio on slurry properties are shown in Table 1.

**Table 1:** Mixing proportions of fly ash and desulfurization gypsum.

No.	Fly Ash: Desulfurization Gypsum	Content of Calcium Carbide Slag (%)	Cement Dosage (%)	Water–Cement Ratio
Figure 1	50:50	15	10	0.65
Figure 2	60:40	15	10	0.65
Figure 3	70:30	15	10	0.65
Figure 4	80:20	15	10	0.65
Figure 5	90:10	15	10	0.65

(1) Slurry Fluidity

**Figure 2:**
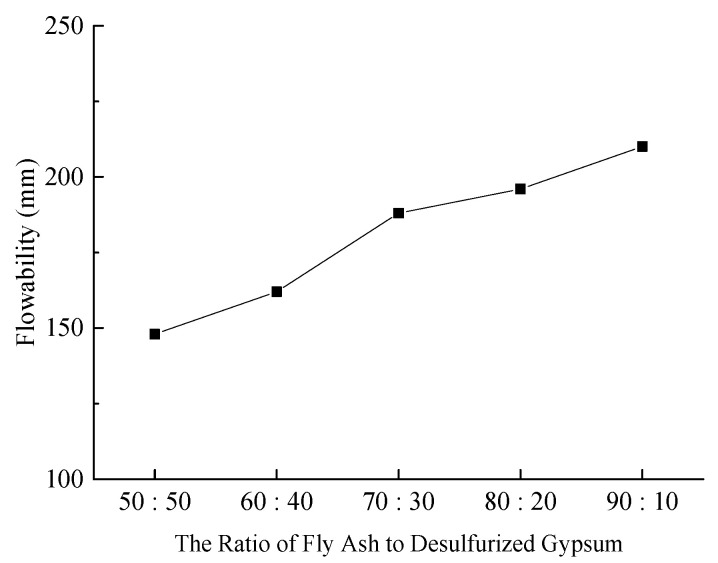
Effect of fly ash and desulfurized gypsum ratios on slurry fluidity.

The fluidity of the slurry significantly affects the pipeline transportation and filling effectiveness of backfill materials. Figure 2 illustrates the slurry fluidity under various experimental conditions.

**Figure 3:**
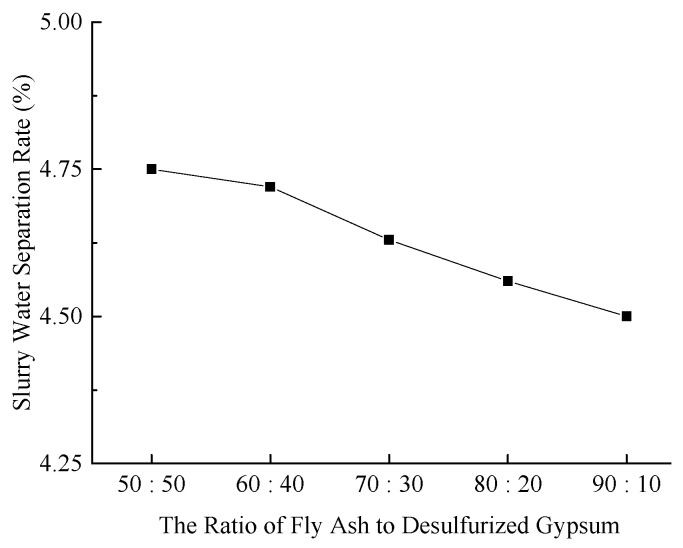
Effect of fly ash-to-desulfurized gypsum ratio on slurry water separation rate.

**Figure 4:**
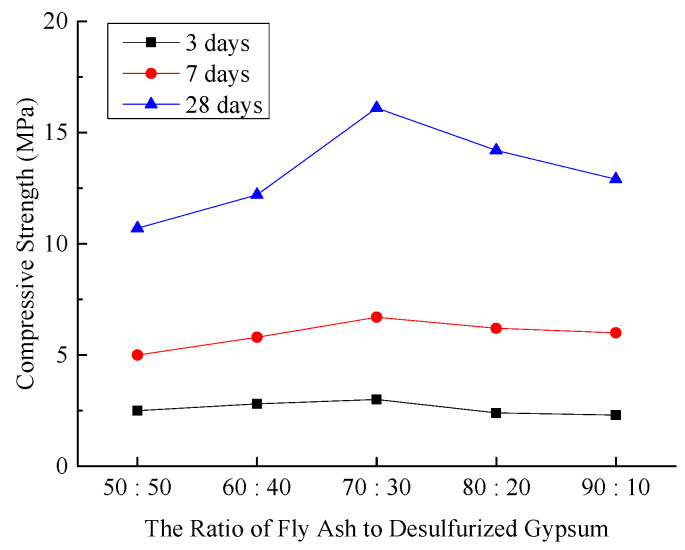
Effect of fly ash-to-desulfurized gypsum ratio on compressive strength of hardened sample.

**Figure 5:**
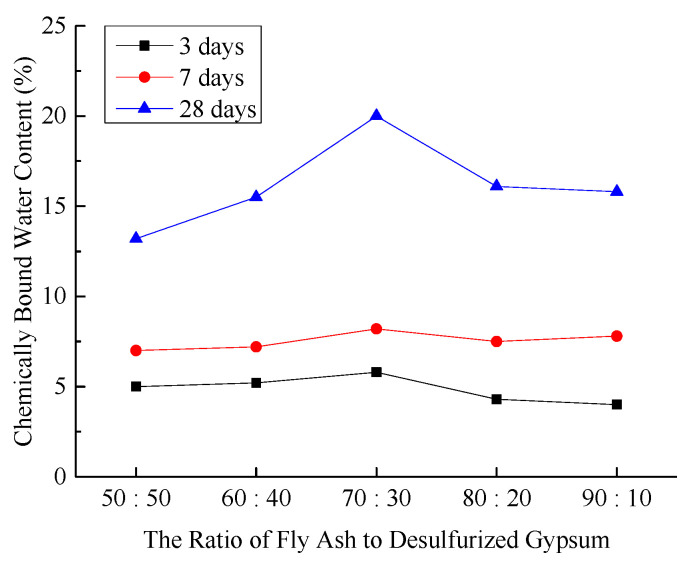
Effect of fly ash-to-desulfurized gypsum ratio on chemically bound water content in hardened sample.

As illustrated in Figure 2, as the proportion of fly ash increases from 50% to 90% (from Figure 1 to Figure 5), the proportion of desulfurized gypsum correspondingly decreases from 50% to 10%, and the fluidity of the slurry continuously improves. This increase in fluidity is attributed to the spherical shape and dense, smooth surface of fly ash particles, which function as a “ball bearing” within the composite slurry, enhancing its flowability. The test results indicate that the fluidity of the slurry in Figure 1 and Figure 2 is relatively low, failing to meet the pipeline transportation requirements for backfill materials. In contrast, the fluidity of the slurry in Figure 3, Figure 4, and Figure 5 exceeds 180 mm, which is sufficient to satisfy the on-site pipeline transportation needs.

(2) Slurry Water Separation Rate

The water separation rate of the slurry is a key indicator for assessing the stability and performance of backfill materials. Figure 3 illustrates the water separation rates of the slurry under various experimental conditions.

As illustrated in Figure 3, the water separation rates of the slurry under all five experimental Figures are below 5%, thus satisfying the on-site performance requirements for backfill materials.

(3) Compressive Strength of Hardened Sample

The compressive strength of the hardened sample, a critical mechanical property for mine filling materials, directly determines its effectiveness in supporting overlying strata. Figure 4 illustrates the compressive strengths of the hardened sample under different experimental conditions.

As depicted in Figure 4, the compressive strength of the hardened sample increases progressively with increasing curing age. Under different experimental conditions, the compressive strength initially rises and then declines as the proportion of fly ash increases. At a curing age of 3 days, the compressive strength curve exhibits minimal variation. At 7 days, the variation in the compressive strength curve becomes more pronounced, and at 28 days, a distinct inflection is observed. When the ratio of fly ash to desulfurized gypsum shifts from 50:50 to 70:30, the compressive strength reaches its maximum. However, as the ratio increases further to 90:10, the compressive strength gradually decreases. This indicates that, initially, desulfurized gypsum primarily functions as a physical filler with minimal impact on strength. As the curing age progresses, desulfurized gypsum activates the reactivity of fly ash, thereby enhancing the strength of the system.

(4) Chemically Bound Water Content in Hardened Sample

The chemically bound water content in the hardened sample acts as a “bridge parameter” linking the microstructures and macroscopic properties of mine filling materials. Its primary function is to balance the requirements for strength, durability, cost, and environmental protection by modulating the degree of the hydration reaction. Figure 5 illustrates the chemically bound water content in the hardened sample under various experimental conditions.

As illustrated in Figure 5, at curing ages of 3 and 7 days, the chemically bound water content in hardened sample of composite cementitious filling materials under different experimental conditions exhibited minimal variation, with Figure 3 showing a slightly higher content than other figures. However, at 28 days, the chemically bound water content in the hardened sample of Figure 3 was significantly higher than that of other Figures. The test results indicate that the reaction rate of the basic fly ash-desulfurized gypsum system is slow, resulting in fewer initial hydration products, and their ratio has minimal impact on the degree of hydration. At 28 days, the pozzolanic effect of fly ash is fully activated, with higher fly ash content leading to more generated hydration products. Desulfurized gypsum plays a role in activating fly ash at later stages. When the fly ash content is high, the proportion of desulfurized gypsum decreases, weakening its activation effect on fly ash and preventing the full utilization of fly ash’s pozzolanic activity, thereby reducing the degree of hydration. Therefore, the chemically bound water content in Figure 4 and Figure 5 gradually decreases.

Taking into account the slurry fluidity, water separation rate, and the compressive strength and chemically bound water content of the hardened sample, the optimal ratio of fly ash to desulfurized gypsum was determined to be 70:30.

#### 3.1.2. Addition of Calcium Carbide Sludge

Based on the findings in Section 3.1.1, the optimal ratio of fly ash to desulfurized gypsum was determined to be 70:30. In this section, the cement dosage is set at 10%, and the water-to-cement ratio is maintained at 0.65. Three experimental sets are designed with varying additional amounts of calcium carbide sludge: 10% in Figure 1, 15% in Figure 2, and 20% in Figure 3. The effect of calcium carbide slag addition amount on slurry performance is shown in Table 2 for the test mix proportions.

(1) Slurry Fluidity

**Figure 6:**
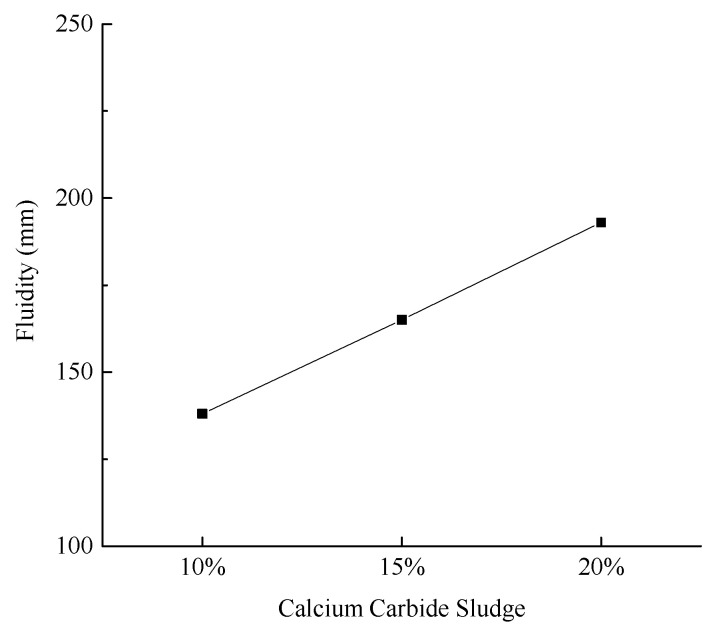
Effect of calcium carbide sludge content on slurry fluidity.

Figure 6 illustrates the slurry fluidity under various experimental conditions.

As illustrated in Figure 6, the fluidity of the slurry increases progressively with the addition of calcium carbide sludge, exhibiting an approximate increase of 30%. Notably, when the calcium carbide sludge content is set at 20%, the slurry achieves optimal fluidity, reaching 193 mm. This level of fluidity is highly suitable for pipeline transportation.

(2) Slurry Water Separation Rate

**Figure 7:**
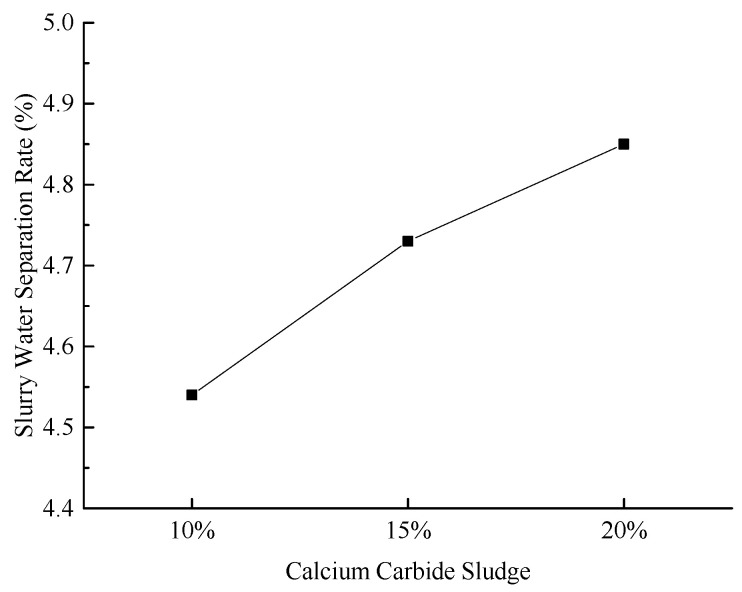
Effect of calcium carbide sludge content on slurry water separation rate.

Figure 7 illustrates the water separation rates of the slurry under various experimental conditions.

As illustrated in Figure 7, the water separation rate of the slurry gradually increases with the addition of calcium carbide sludge, although the increase remains relatively modest. Notably, the water separation rate consistently remains below 5%, indicating no significant adverse impact on the slurry’s stability. Experimental results reveal that the incorporation of calcium carbide sludge enhances the slurry’s fluidity. The OH^−^ ions facilitate the formation of gels by reacting with the aluminum oxide and silicon dioxide in the fly ash. Simultaneously, an electrophoretic effect occurs, causing partial precipitation in the system and consequently increasing the water separation rate.

(3) Compressive Strength of Hardened Sample

**Figure 8:**
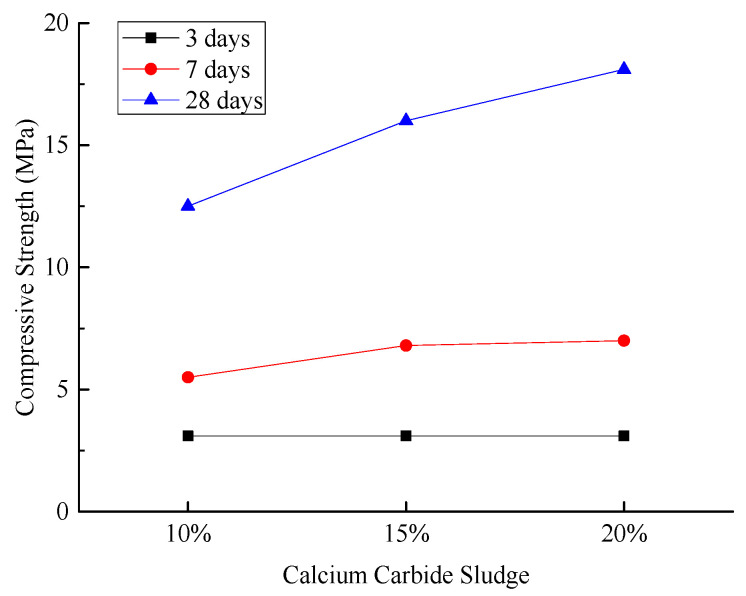
Effect of calcium carbide sludge content on compressive strength of hardened sample.

Figure 8 illustrates the compressive strengths of the hardened sample under different experimental conditions.

As illustrated in Figure 8, the compressive strength of the hardened sample at the 3-day curing age remains nearly unchanged with increasing calcium carbide sludge content. At the 7-day curing age, the strength exhibits a slight increase, albeit with a moderate growth margin. Notably, at the 28-day curing age, the compressive strength shows a significant enhancement. These findings suggest that the development of compressive strength in the cementitious material system is a slow-growth process, and a higher calcium carbide sludge content facilitates the gradual increase in the system’s compressive strength.

Mechanistic analysis reveals that the incorporation of calcium carbide sludge enhances the internal structure of the cementitious system. Specifically, Ca(OH)_2_ rapidly increases the alkalinity of the system, providing a highly alkaline environment that activates fly ash. The hydrated Ca(OH)_2_ diffuses to the periphery of the fly ash glassy spheres, effectively disrupting their structure and releasing active Al_2_O_3_ and SiO_2_. In this alkaline environment, Ca^2+^ reacts with Al-O and Si-O bonds to form calcium silicate hydrate (C-S-H) and calcium aluminate hydrate (C-A-H) gels. These gels densify the microstructure of the hardened sample, thereby enhancing their compressive strength [38,39].

(4) Chemically Bound Water Content in Hardened Sample

**Figure 9:**
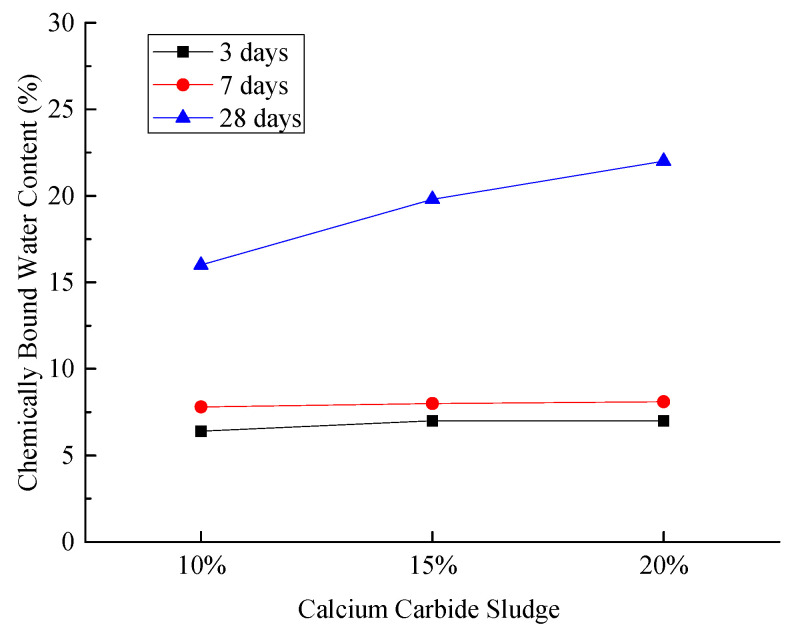
Effect of calcium carbide sludge content on chemically bound water content in hardened sample.

Figure 9 illustrates the chemically bound water content in the hardened sample under various experimental conditions.

As illustrated in Figure 9, the chemically bound water content in the hardened sample shows minimal growth with increasing calcium carbide sludge content and remains relatively low at curing ages of 3 and 7 days. In contrast, a significant increase is observed at a curing age of 28 days. This indicates that during the early hydration stages, the dissolution of Al-O and Si-O bonds in fly ash occurs slowly under alkaline conditions, leading to a low degree of hydration and minimal chemically bound water. Consequently, the effect of calcium carbide sludge is not pronounced. However, at 28 days, test groups with higher calcium carbide sludge content exhibit enhanced activation of fly ash, resulting in a greater degree of hydration, more hydration products, and a higher chemically bound water content [40,41].

#### 3.1.3. Cement Content

Based on the aforementioned research results, the optimal ratio of fly ash to desulfurized gypsum is determined to be 70:30, and the optimal content of calcium carbide sludge is established at 20%. On this basis, this section sets the water-to-cement ratio at 0.65 and designs three experimental groups with cement contents of 5%, 10%, and 15%, respectively. The test mix proportions for the effect of cement dosage on slurry performance are shown in Table 3.

(1) Slurry Fluidity

**Figure 10:**
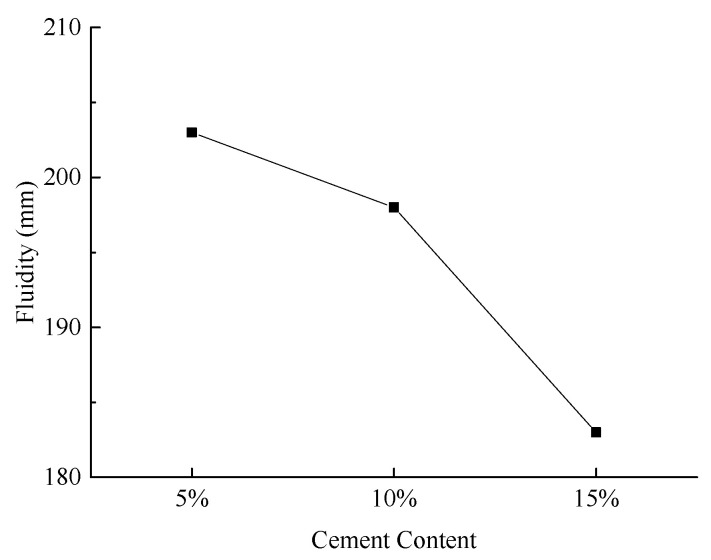
Effect of cement content on slurry fluidity.

Figure 10 illustrates the slurry fluidity under various experimental conditions.

As illustrated in Figure 10, the slurry fluidity decreases with increasing cement content. This decrease is attributed to the higher water demand associated with increased cement content, which results in a relative reduction in free water within the system, thereby diminishing the slurry’s fluidity.

(2) Slurry Water Separation Rate

**Figure 11:**
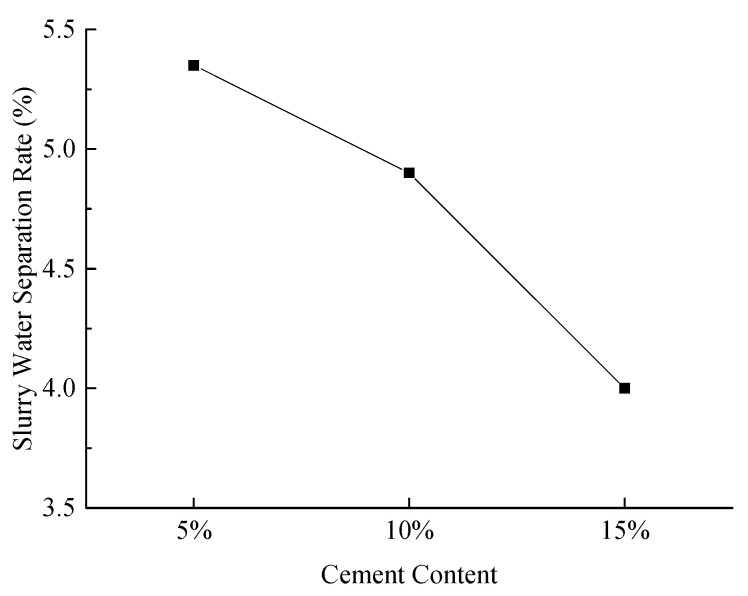
Effect of cement content on slurry water separation rate.

Figure 11 illustrates the water separation rates of the slurry under various experimental conditions.

As illustrated in Figure 11, the water separation rate of the slurry decreases with increasing cement content. This decrease is attributed to the rapid hydration of cement, which solidifies fly ash and desulfurized gypsum together, thereby enhancing the stability of the system.

(3) Compressive Strength of Hardened Sample

**Figure 12:**
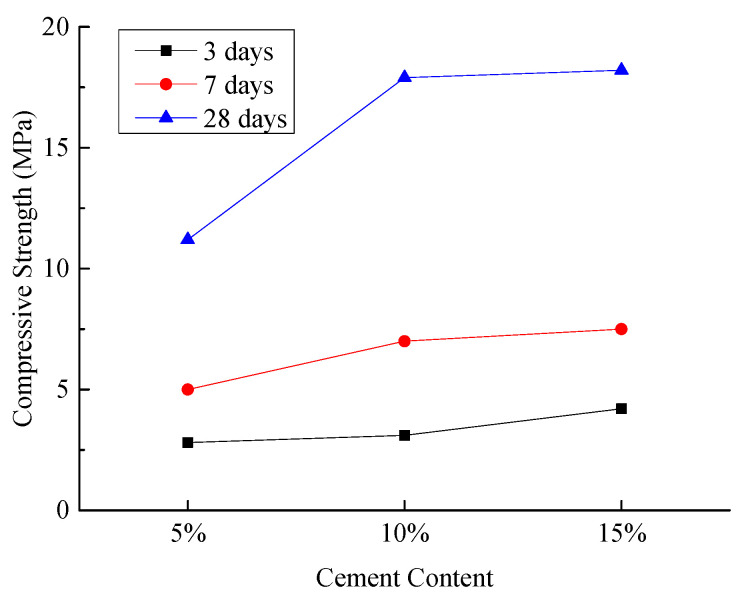
Effect of cement content on compressive strength of hardened sample.

Figure 12 illustrates the compressive strengths of the hardened sample under different experimental conditions.

As illustrated in Figure 12, increasing the cement content from 5% to 10% results in an increase in the compressive strength of the hardened sample at all curing ages, with a particularly significant increase observed at 28 days. Further increasing the cement content from 10% to 15% continues to enhance the compressive strength, although the rate of increase slows at 28 days. When the cement content is set at 10%, the compressive strength of the hardened sample reaches 17.69 MPa, meeting the on-site construction requirements. The test results indicate that as the cement content increases, the strength of the cementitious materials gradually rises. When the cement content exceeds 10%, the late-stage compressive strength of the composite cementitious materials continues to increase, albeit at a decreasing rate. This suggests that an appropriate cement content fully activates the active minerals in the composite cementitious materials through alkaline activators. The early compressive strength of the composite cementitious materials is primarily contributed by the active minerals in cement clinker, which exhibit high cementitious activity and rapid hydration reactions, forming hydration products in the initial mixing stage. The late-stage compressive strength is mainly contributed by the active minerals in fly ash and the unreacted active minerals in clinker. The alkaline environment formed by the reaction of cement clinker and the addition of alkaline activators provides the necessary alkalinity for the active components in fly ash, enabling these components to hydrate slowly in the later stages and significantly contribute to the late strength [42,43].

(4) Chemically Bound Water Content in Hardened Sample

**Figure 13:**
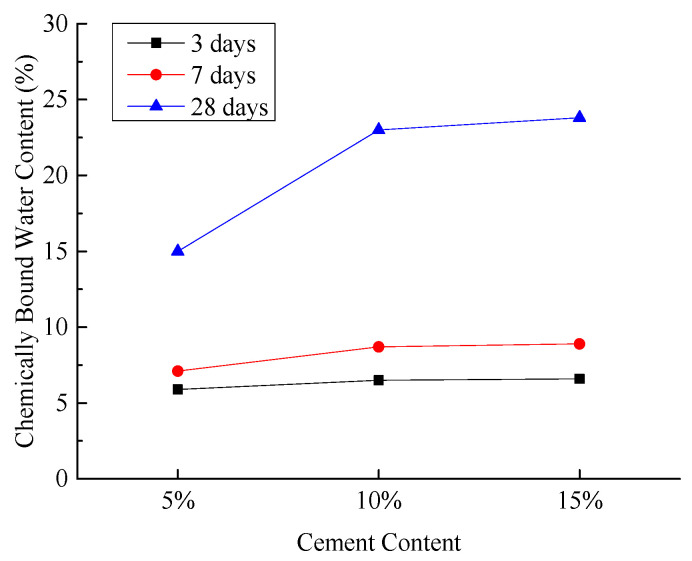
Effect of cement content on chemically bound water content in hardened sample.

Figure 13 illustrates the chemically bound water content in the hardened sample under various experimental conditions.

As illustrated in Figure 13, the chemically bound water content in the hardened sample increases with curing age under different experimental conditions. When the cement content increases from 5% to 10%, the chemically bound water content at the 28-day curing age shows a significant increase. However, when the cement content increases from 10% to 15%, the increase in chemically bound water content at 28 days is less pronounced. The test results indicate that the early hydration reaction of ordinary Portland cement and fly ash is relatively slow. As the curing age increases, cement hydration generates more Ca(OH)_2_, creating a more favorable alkaline environment for the activation of fly ash and desulfurized gypsum, leading to the formation of additional hydration products. At 28 days, the cement contents of 10% and 15% provide more C_2_S and C_3_S, which accelerate the growth of chemically bound water in the test groups. As curing continues, the late-stage hydration of C_2_S progresses, causing the chemically bound water content in both test groups to converge and increase gradually [44,45].

#### 3.1.4. Water–Cement Ratio

Based on the previous research results, the optimal ratio of fly ash to desulfurized gypsum was determined to be 70:30, with the optimal addition amounts of calcium carbide sludge and cement set at 20% and 10%, respectively. On this basis, three experimental sets were designed in this section, with water-to-cement ratios of 0.55, 0.65, and 0.75. It should be noted that the mixing water used for composite cementitious backfill materials shall be purified and desalinated water. The test mix proportions for investigating the effect of water–cement ratio on slurry performance are shown in Table 4.

(1) Slurry Fluidity

**Figure 14:**
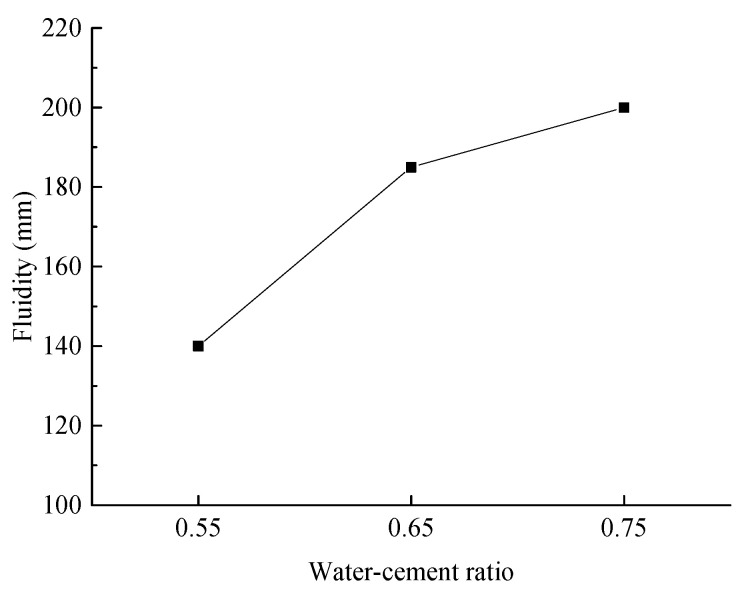
Effect of water-to-cement ratio on slurry fluidity.

Figure 14 illustrates the slurry fluidity under various experimental conditions.

As illustrated in Figure 14, the slurry fluidity increases significantly and continuously with the water-to-cement ratio rising from 0.55 to 0.75. At a water-to-cement ratio of 0.55, the slurry flow value is 140 mm, which is insufficient for pipeline transportation. In contrast, at water-to-cement ratios of 0.65 and 0.75, the slurry flow values exceed 180 mm, satisfying the requirements for on-site pipeline transportation.

(2) Slurry Water Separation Rate

**Figure 15:**
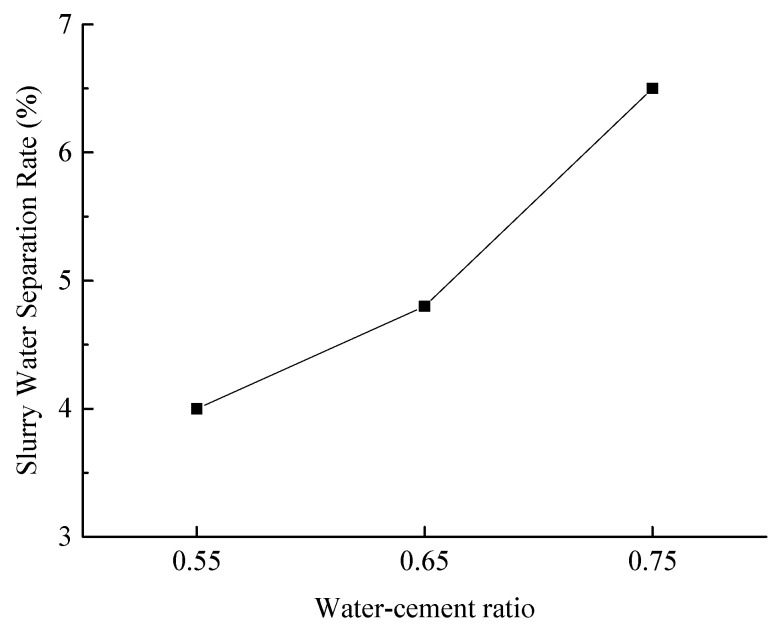
Effect of water-to-cement ratio on slurry water separation rate.

Figure 15 illustrates the water separation rates of the slurry under various experimental conditions.

As illustrated in Figure 15, the water separation rate of the slurry increases progressively with the water-to-cement ratio. At water-to-cement ratios of 0.55 and 0.65, the water separation rates are both below 5%, indicating satisfactory slurry stability. However, at a water-to-cement ratio of 0.75, the water separation rate rises to 6.43%, suggesting diminished slurry stability.

Taking into account both the fluidity and water separation rate of the slurry, the optimal water-to-cement ratio is determined to be 0.65.

### 3.2. Microstructural Characteristics and Hydration Mechanism of Composite Cementitious Backfill Materials

Based on the mix proportion parameters established in the preceding section, the base group has a fly ash-to-desulfurized gypsum ratio of 70:30. In the additive group, the calcium carbide sludge content is 20%, the cement content is 15%, and the water-to-cement ratio is set at 0.65. The hydration process and reaction mechanism of the cementitious materials were analyzed microscopically using X-ray diffraction (XRD) and scanning electron microscopy (SEM) techniques.

#### 3.2.1. XRD Analysis of Composite Cementitious Backfill Materials

**Figure 16:**
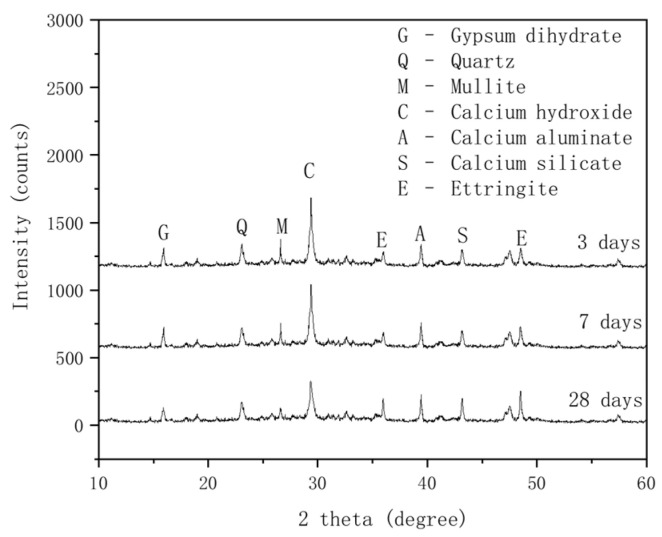
XRD patterns of hydration products in cementitious materials.

Figure 16 illustrates the XRD patterns of composite cementitious backfill materials at curing ages of 3, 7, and 28 days.

As illustrated in Figure 16, at a curing age of 3 days, the hydration reaction begins to take place. The diffraction peaks of Ca(OH)_2_, mullite, and quartz in the composite cementitious backfill materials are relatively distinct, with minor peaks of ettringite, calcium silicate, and calcium aluminate also present. Ettringite plays a primary role in the early strength of the backfill material. The Ca(OH)_2_ diffraction peak remains prominent at all ages, but as the curing age extends to 7 days, Ca(OH)_2_ is consumed by hydration reactions, leading to a decrease in peak intensity. The peaks of mullite and quartz also decrease, as Al_2_O_3_ and SiO_2_ in fly ash react with Ca(OH)_2_ (a cement hydration product) to form C-A-H and C-S-H gels. When the curing age increases to 28 days, the Ca(OH)_2_ diffraction peak continues to decline. The pozzolanic effect of fly ash is further activated, and the hydration reaction consumes substantial Ca(OH)_2_, generating more C-A-H and C-S-H gels, which enhance the compressive strength of the backfill material.

With increasing curing age, the diffraction peak of gypsum (calcium sulfate dihydrate) begins to decrease: the decline is minimal at 7 days but becomes pronounced at 28 days. In the later stages, the activity of fly ash is secondarily activated by desulfurized gypsum, leading to significant consumption of desulfurized gypsum. At 28 days, the peaks of calcium silicate and ettringite increase significantly. Part of the calcium aluminate reacts with gypsum to form ettringite crystals, consuming calcium aluminate and Ca(OH)_2_ while increasing the content of hydration products, particularly ettringite crystals. The hydration products are primarily composed of C-S-H gel and ettringite, which contribute to the strength of the fly ash composite cementitious materials [46,47,48].

#### 3.2.2. SEM Analysis of Composite Cementitious Backfill Materials

**Figure 17:**
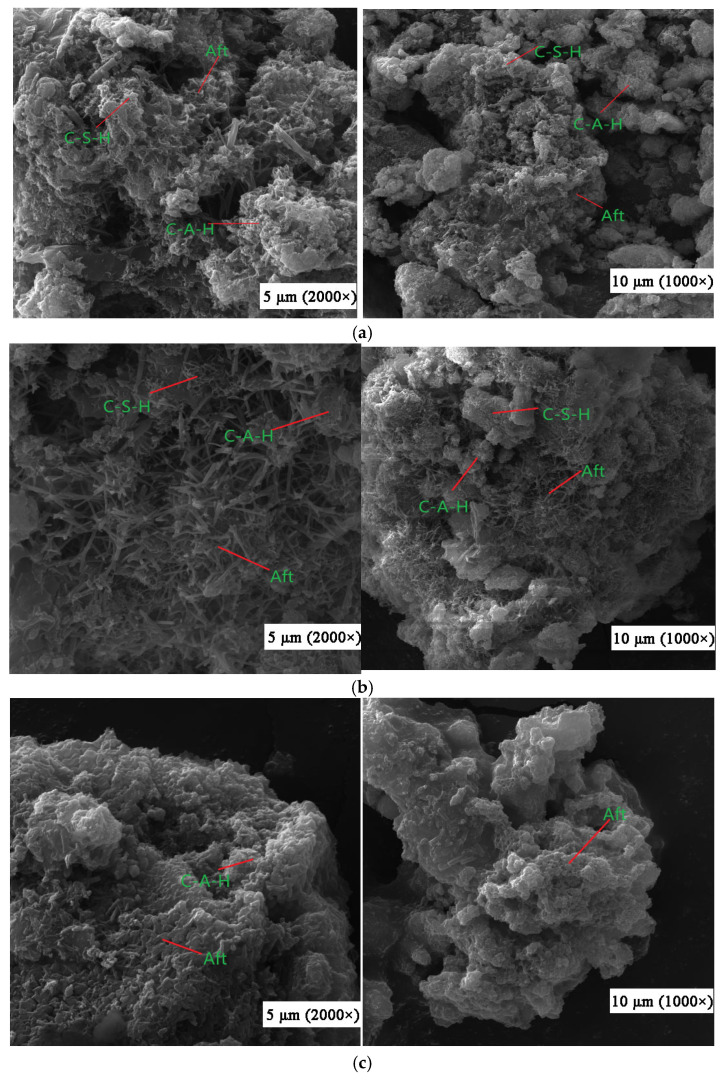
SEM images of hydration products in composite cementitious filling materials at (**a**) 3 days of hydration; (**b**) 7 days of hydration; (**c**) 28 days of hydration.

Figure 17 illustrates the SEM results of composite cementitious backfill materials.

From Figure 17a, it is evident that the composite cementitious backfill materials exhibit surface erosion at a hydration age of 3 days. At this stage, the vitreous structure of fly ash is disrupted, leading to the gradual cleavage of Si-O and Al-O bonds on the fly ash surface. The presence of white flocculent substances indicates that the reactive components of fly ash have initiated reactions. Specifically, Ca(OH)_2_ reacts with SiO_2_ in fly ash to form C-S-H gel (calcium silicate hydrate), while Ca(OH)_2_ reacts with Al_2_O_3_ to generate C-A-H gel (calcium aluminate hydrate). The interlaced acicular substances observed are ettringite (Aft, calcium sulfoaluminate hydrate) formed by the reaction of Al_2_O_3_ with SO_4_^2−^ and Ca(OH)_2_, which significantly contributes to the early strength development of the composite cementitious backfill materials [49,50].

As the hydration process progresses and under the dual activation effect of Ca(OH)_2_ and desulfurized gypsum, Figure 17b shows that at 7 days of hydration, the surface erosion of the composite cementitious backfill materials becomes more pronounced. The white flocculent substances increase, and hydration products accumulate. A continuous network structure gradually forms on the surface of fly ash particles, where alumina and silica in the fly ash vitreous body are increasingly dissolved and participate in hydration reactions. Many individual erosion points on the particle surface merge into continuous areas, and more granular hydration products appear on the surface of the cementitious materials. The acicular ettringite crystals grow longer, denser, and interlace more intensively. Under the alkaline conditions provided by the hydration of carbide slag and cement, the vitreous structure of fly ash gradually disintegrates. Driven by desulfurized gypsum, the pozzolanic activity of fly ash is continuously activated, leading to an increasing number of hydration reactions.

At 28 days of hydration, Figure 17c shows that the vitreous structure of fly ash is almost entirely eroded, and its surface is completely covered by hydration products. The morphological characteristics of fly ash become indistinct. In the cementitious materials, acicular ettringite crystals interlock to form an integral structure with fewer voids and a denser texture, enhancing the compactness and strength of the backfill materials. Meanwhile, hexagonal plate-like crystals of desulfurized gypsum can be seen precipitating and interpenetrating the cementitious material system, further increasing the strength of the system.

**Figure 18:**
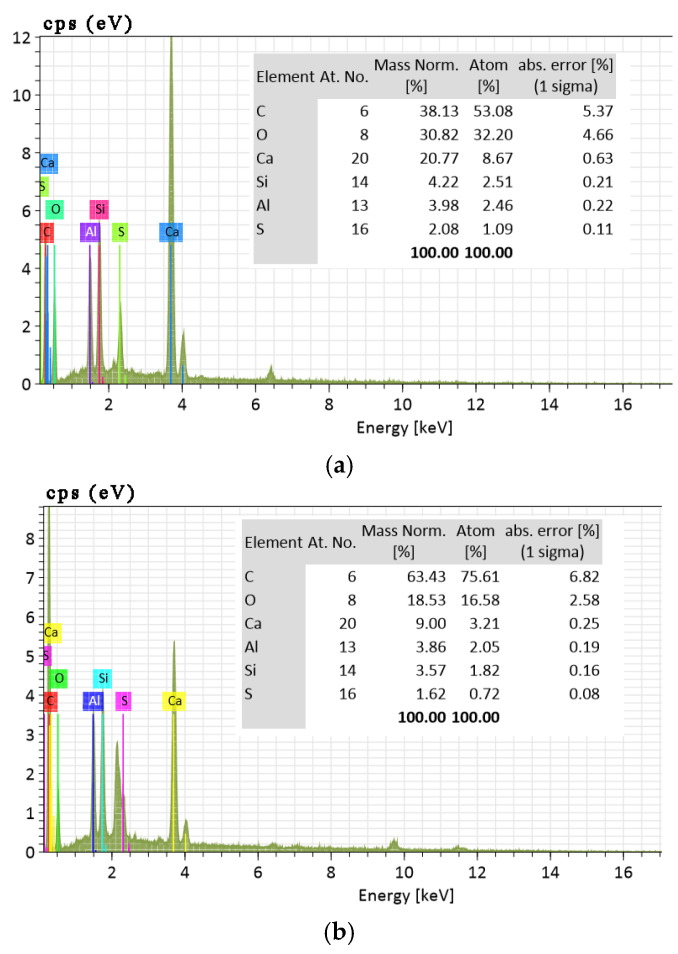

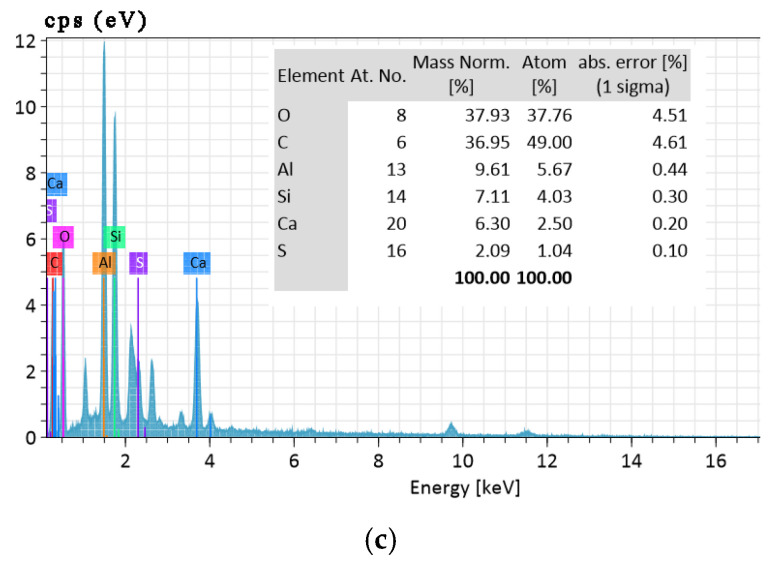
EDS spectra of hydration products in composite cementitious backfill materials at (**a**) 3 days of hydration; (**b**) 7 days of hydration; (**c**) 28 days of hydration.

The EDS spectra of the hydration products of composite cementitious backfill materials are illustrated in Figure 18.

As illustrated in Figure 18a, at a hydration age of 3 days, the peaks of Al and Si are relatively low, indicating that the activity of fly ash has not yet been fully activated. The Al-Si bonds in fly ash are tightly bonded, while the peak of Ca is relatively high. The initial reaction is slow, with OH^−^ ions from Ca(OH)_2_ playing a dominant role in accelerating the breakage of Al-Si bonds in fly ash. At 7 days of hydration (Figure 18b), the Ca peak gradually decreases, suggesting the formation of more calcium silicate and calcium aluminate gels. As shown in Figure 18c, by 28 days of hydration, the hydration reaction is essentially complete, with ettringite crystals being the primary reaction product. The peaks of Al and Si increase significantly, as the system consumes Ca^2+^ to generate a large amount of ettringite.

### 3.3. Hydration Mechanism Analysis

The crystalline phases and micro-morphologies of composite cementitious backfill materials at different curing ages were systematically analyzed using XRD (X-ray Diffraction) and SEM (Scanning Electron Microscopy) techniques to investigate the hydration process of the fly ash-desulfurized gypsum-calcium carbide sludge–cement composite system.

During the hydration of cement, a significant amount of Ca(OH)_2_ colloidal particles are generated. Similarly, calcium carbide sludge forms substantial Ca(OH)_2_ colloidal particles upon contact with water. When fly ash is mixed with water, a water film forms on its surface. Upon contact with Ca(OH)_2_, Ca^2+^ and OH^−^ ions penetrate the water film on the fly ash surface, forming an alkaline film containing OH^−^ and Ca^2+^. Once the alkaline film is established, the fly ash surface begins to corrode, leading to the partial rupture of Si-O and Al-O bonds on the fly ash particle surface and initiating the pozzolanic reaction, as follows [51,52]:

(2) $$SiO_{2}+mCa{\left(OH\right)}_{2}+nH_{2}O=mCaO\cdot SiO_{2}\cdot nH_{2}O$$

(3) $${Al}_{2}O_{3}+mCa{\left(OH\right)}_{2}+nH_{2}O=mCaO\cdot {Al}_{2}O_{3}\cdot nH_{2}O$$

(4) $$mCaO∙{Al}_{2}O_{3}∙nH_{2}O+CaSO_{4}∙2H_{2}O=mCaO∙{Al}_{2}O_{3}∙CaSO_{4}∙(n+2)H_{2}O$$

As the system experiences a progressive increase in both free water and chemically bound water, the concentration of OH^−^ ions within the alkaline microenvironment at the fly ash surface correspondingly rises. Concurrently, these ions infiltrate the interior through interstices among surface hydrates, instigating corrosion of the fly ash vitreous phase and promoting the deepening of the pozzolanic reaction. With the increase in [OH^−^] and [Ca^2+^] molarities in the cementitious matrix, the hydration kinetics of fly ash accelerates in a stepwise manner. Initially, trace amounts of ettringite are formed, while cement hydration contributes calcium silicate hydrate (C-S-H) gel, which supports early strength development. However, as deduced from the early-age compressive strength data presented in Chapter 4, the cementitious grouting material exhibits suboptimal early strength. This is attributed to the strong electrostatic adsorption of Ca^2+^ and OH^−^ by colloidal nuclei within the Ca(OH)_2_ particle matrix, which imposes a thermodynamic constraint on ion availability—particularly for Ca^2+^—in the alkaline interfacial solution. The hydration products, primarily existing as CaO·SiO_2_·nH_2_O (C-S-H) and CaO·Al_2_O_3_·nH_2_O (C-A-H) gels (see reactions in Equations (2) and (3)), further exacerbate ion sequestration through their inherent sorption capacity. Given that C-S-H and C-A-H constitute the fundamental strength-contributing phases in fly ash-based systems, the retarded hydration kinetics result in diminished early-age mechanical performance. As curing progresses, the accumulating C-A-H phase engages in a secondary reaction with dihydrate gypsum to form ettringite (CaO·Al_2_O_3_·3CaSO_4_·(n + 2)H_2_O), as delineated in Equation (4), driving progressive strength gain through the formation of a denser crystalline network [53,54].

Based on the foregoing analysis, the hydration reaction of the fly ash-desulfurized gypsum-calcium carbide sludge–cement composite system is fundamentally characterized as a synergistic process that integrates cement clinker hydration and the pozzolanic activation of fly ash by desulfurized gypsum and calcium carbide sludge. This process can be delineated into three distinct stages [55,56]:

Stage I: Activation of Silicon–Aluminum Bonds in Fly Ash

Cement clinker hydration releases calcium hydroxide, while calcium carbide sludge introduces additional Ca(OH)_2_, creating a highly alkaline microenvironment (pH > 12.5) rich in OH^−^ ions. These OH^−^ ions initiate nucleophilic attacks on the Si-O and Al-O bonds of fly ash vitreous particles, facilitating the dissolution of reactive SiO_2_ and Al_2_O_3_ species. The dissolved SiO_2_ reacts with Ca(OH)_2_ to form calcium silicate hydrate (C-S-H) gel, while Al_2_O_3_ generates calcium aluminate hydrate (C-A-H) gel. Concurrently, C-A-H gel reacts with sulfate ions (SO_4_^2−^) from desulfurized gypsum and excess Ca(OH)_2_ to precipitate ettringite (3CaO·Al_2_O_3_·3CaSO_4_·32H_2_O). These hydration products—C-S-H, C-A-H, and ettringite—deposit on particle surfaces to form a dense encapsulation layer, as confirmed by in situ SEM observations [57].

Stage II: Progressive Hydration and Structural Erosion

The continuous erosion of the fly ash vitreous network occurs as Ca^2+^, OH^−^, and SO_4_^2−^ ions diffuse through the porous encapsulation layer. The needle-like morphology of ettringite crystals enhances structural porosity, thereby accelerating ion transport and promoting deeper dissolution of aluminosilicate phases. This stage is kinetically governed by multiple factors, including the Al/Si ratio of the vitreous phase, reaction temperature (activation energy: 55–65 kJ/mol), and fly ash reactivity. Isothermal calorimetry reveals a secondary exothermic peak at approximately 14 days, attributed to the accelerated pozzolanic reaction. XRD analysis confirms the gradual consumption of amorphous phases and the formation of a heterogeneous hydration product matrix [58].

Stage III: Crystalline Growth and Strength Development

The sustained structural degradation of fly ash particles expands the reactive interface, enabling alkaline solutions to penetrate deeper into particle cores. Continuous hydration generates an increasingly dense microstructure characterized by interlocking C-S-H/C-A-H gels and ettringite crystals. Mercury intrusion porosimetry demonstrates a reduction in median pore diameter from 120 nm at 3 days to 45 nm at 28 days, correlating with the development of mechanical strength. Compressive strength data follow a quadratic growth model, reflecting the progressive densification of the hydration product network. Thermogravimetric analysis (TGA) confirms a 25% increase in chemically bound water content over the same period, further validating the hydration progression [59].

### 3.4. Field Test

**Figure 19:**
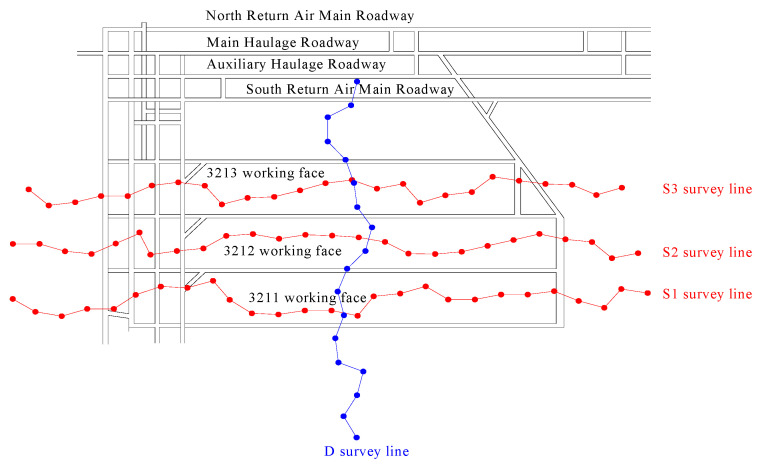
Layout of surface monitoring points.

After the working face is mined, a retaining wall composed of “I-beams + metal mesh + air duct cloth” is constructed at the end of the working face. The prepared composite cemented backfill material is then filled into the goaf through pipelines. The No. 3 coal seam has an average buried depth of 550 m and an average thickness of 2.5 m. According to the relevant regulations of the Coal Mine Surveying Code, when the mining depth exceeds 300 m, the working point spacing of the surface movement observation station should be 20–25 m. In this study, based on the topographic conditions of the surface corresponding to the three test working faces (3211, 3212, and 3213), the point spacing is set to 25 m. Specifically, a monitoring curve is arranged in the middle of each working face along the strike direction, labeled as S1, S2, and S3, respectively. Additionally, a monitoring curve is arranged along the dip direction, labeled as D. The S1 survey line comprises 27 measuring points, the S2 survey line comprises 26 measuring points, the S3 survey line comprises 25 measuring points, and the D survey line comprises 18 measuring points. The corresponding range of the working face surface and the layout of the surface measuring points are depicted in Figure 19.

**Figure 20:**
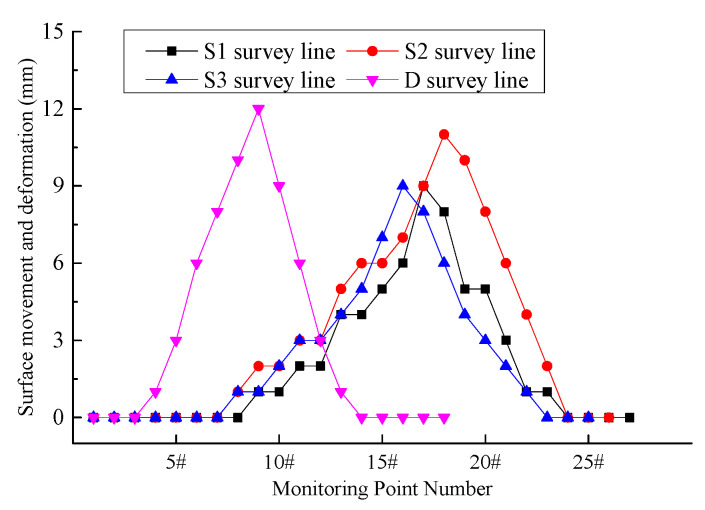
Surface movement and deformation monitoring curves.

Figure 20 illustrates the surface movement and deformation monitoring data.

As illustrated in Figure 20, the maximum surface subsidence along the S1 survey line is 9 mm, along the S2 survey line is 11 mm, along the S3 survey line is 9 mm, and along the D survey line is 12 mm. The field monitoring data indicate that the surface movement and deformation are minimal, falling within the Grade I range specified in the Regulations for Coal Mining Under Buildings, Railways, and Water Bodies (hereinafter referred to as the “Three Under” Mining Regulations). This level of movement meets the protection requirements for surface buildings and structures.

## 4. Conclusions

This study utilizes coal-based solid waste materials, including fly ash, desulfurized gypsum, and carbide sludge, as the foundation and incorporates cement, water, and other raw materials to develop a novel composite cementitious backfill material. Field tests were conducted at Xinyuan Coal Mine, yielding the following principal conclusions:

(1) The hydration process of cementitious backfill materials is characterized by the synergistic interaction among fly ash, desulfurized gypsum, and carbide sludge. The calcium hydroxide in carbide sludge rapidly elevates the alkalinity of the system, facilitating the depolymerization of the glassy structure in fly ash. This significantly enhances the early pozzolanic activity of fly ash and accelerates the formation of C-S-H gels. The addition of cement provides early strength support to the material system. The ettringite and C-S-H gels generated during cement hydration form the strength framework. Desulfurized gypsum, on the other hand, continuously undergoes secondary reactions with the hydration products of fly ash in the later stages. Through the continuous crystallization and growth of ettringite, it ensures the steady increase in material strength.
(2) The optimal mix proportion of cementitious backfill materials was determined through single-variable method tests as follows: The mass ratio of fly ash to desulfurized gypsum is 70:30, the carbide sludge content is 20%, the cement content is 15%, and the water–binder ratio is 0.65. Under this proportion, the fluidity of the composite cementitious backfill materials ranges from 180 to 220 mm, the water bleeding rate within 6 h is less than 5%, and the 28-day compressive strength reaches 17.69 MPa. In addition, XRD and SEM analyses indicate that the hydration products of the system under this mix proportion have high crystallinity and a dense pore structure, providing a guarantee for long-term stability.
(3) The results of on-site industrial tests indicate that after the goafs are filled with the new composite cementitious backfill materials, the maximum surface subsidence is only 12 mm, which is far lower than the Grade I settlement standard (≤30 mm) specified in the Regulations for Mining Under Buildings, Railways and Water Bodies, fully meeting the protection requirements for surface buildings and structures. As the raw materials mainly consist of coal-based solid waste materials such as fly ash, desulfurized gypsum, and carbide sludge, the filling cost is effectively reduced, achieving the unity of environmental and economic benefits. Subsequent research could focus on the long-term durability assessment of the materials under complex geological conditions (such as high-confined water and highly corrosive strata). In the future, tests on chloride salt erosion, sulfate erosion, and freeze–thaw cycles could be carried out. Meanwhile, exploring nanomaterial modification techniques to further enhance the mechanical properties and impermeability of the materials, expanding their application potential in deep mining fields.
(4) It should be noted that the experimental results presented in this study are based on a limited number of samples due to constraints in material availability. While the findings provide valuable insights into the performance of the developed backfill material, further validation with a larger sample size is recommended to ensure statistical reliability and support industrial-scale decision-making. This study can be considered a preliminary investigation, and future work should focus on expanding the dataset to enhance the robustness of the conclusions.

## Figures and Tables

**Table 2:** Test mix proportions of calcium carbide slag dosage.

No.	Fly Ash: Desulfurization Gypsum	Content of Calcium Carbide Slag (%)	Cement Dosage (%)	Water–Cement Ratio
Figure 1	70:30	10	10	0.65
Figure 2	70:30	15	10	0.65
Figure 3	70:30	20	10	0.65

**Table 3:** Test mix proportions of cement dosage.

No.	Fly Ash: Desulfurization Gypsum	Content of Calcium Carbide Slag (%)	Cement Dosage (%)	Water–Cement Ratio
Figure 1	70:30	20	5	0.65
Figure 2	70:30	20	10	0.65
Figure 3	70:30	20	15	0.65

**Table 4:** Test mix proportions for water–cement ratio experiments.

No.	Fly Ash: Desulfurization Gypsum	Content of Calcium Carbide Slag (%)	Cement Dosage (%)	Water–Cement Ratio
Figure 1	70:30	20	10	0.55
Figure 2	70:30	20	10	0.65
Figure 3	70:30	20	10	0.75

## Data Availability

The original contributions presented in this study are included in the article. Further inquiries can be directed to the corresponding authors.
